# Survival analysis of Chagas disease patients, beneficiaries of social security and social assistance in Brazil, 1942–2016

**DOI:** 10.1590/1980-549720240020

**Published:** 2024-05-17

**Authors:** Jean Ezequiel Limongi, Keile Aparecida Resende Santos, Izabela Lima Perissato, Rogério de Melo Costa Pinto, Tânia Maria da Silva Mendonça, Ana Elisa Madalena Rinaldi

**Affiliations:** I Universidade Federal de Uberlândia, Collective Health Program – Uberlândia (MG), Brazil.; II Instituto Nacional do Seguro Social – Uberlândia (MG), Brazil.; III Universidade Federal de Uberlândia, School of Mathematics – Uberlândia (MG), Brazil.; IV Universidade Federal de Uberlândia, School of Medicine – Uberlândia (MG), Brazil.

**Keywords:** Chagas disease, Survival analysis, Cohort studies, Social welfare, Social security, Social support, Doença de Chagas, Análise de sobrevida, Estudos de coorte, Seguridade social, Previdência social, Apoio social

## Abstract

**Objective:**

To analyze the survival of patients with Chagas disease, beneficiaries of social security and social assistance, in Brazil, from 1942 to 2016.

**Methods:**

This is a retrospective cohort study with data from the Brazilian Ministry of Social Security. The event of interest was death, and the survival functions were estimated by the Kaplan-Meier and Cox regression methods.

**Results:**

In the period “onset of the disease until death”, women (HR=0.54; 95%CI 0.43–0.53) and receiving social security benefits (HR=0.13; 95%CI 0.11–0.23) were associated with longer survival. Lower survival was associated with the cardiac form of the disease (HR=2.64; 95%CI 2.23–3.12), living in a rural area (HR=1.23; 95%CI 1.14–1.21), and manifestation of the disease between the years 2000 and 2016 (HR=5.32; 95%CI 4.74–5.93). Likewise, in the period “work disability until death”, women (HR=0.51; 95%CI 0.41–0.52) and receiving social security benefits (HR=0.24; 95%CI 0,14–0.45) were associated with longer survival, as well as the cardiac form of the disease (HR=1.95; 95%CI 1.83–2.13), living in a rural area (HR=1.31; 95%CI 1.21–1.54), and manifestation of the disease between 2000 and 2016 (HR=1.53; 95%CI 1.33–1.71) were associated with lower survival.

**Conclusion:**

The main predictors of mortality and survival of patients with Chagas disease who receive social security and assistance benefits in Brazil were presented. These findings can guide the definition of priorities for follow-up actions by Primary Health Care, currently recommended for the longitudinal management of the disease.

## RESUMO

**Objetivo:**

Analisar a sobrevida de portadores da doença de Chagas, beneficiários da previdência e da assistência social no Brasil, 1942-2016.

**Métodos:**

Estudo de coorte retrospectivo com dados do Ministério da Previdência Social. O evento de interesse foi o óbito, e as funções de sobrevida foram estimadas pelos métodos Kaplan-Meier e de regressão de Cox.

**Resultados:**

No período “*início da doença até o óbito*”, o sexo feminino (HR=0,54; IC95% 0,43–0,53) e recebimento de benefícios previdenciários (HR=0,13; IC95% 0,11–0,23) foram associados a maior sobrevida. A menor sobrevida esteve associada à forma cardíaca da doença (HR=2,64; IC95% 2,23–3,12), residência em zona rural (HR=1,23; IC95% 1,14–1,21) e manifestação da doença entre os anos de 2000 e 2016 (HR=5,32; IC95% 4,74–5,93). Da mesma forma, no período “*incapacidade laboral até o óbito*”, o sexo feminino (HR=0,51; IC95% 0,41–0,52) e o recebimento de benefícios previdenciários (HR=0,24; IC95% 0,14–0,45) foram associados a maior sobrevida, assim como forma cardíaca da doença (HR=1,95; IC95% 1,83–2,13), residência em zona rural (HR=1,31; IC95% 1,21–1,54) e manifestação da doença entre os anos de 2000 e 2016 (HR=1,53; IC95% 1,33–1,71) associaram-se a menor sobrevida.

**Conclusão:**

Os principais preditores de mortalidade e sobrevida de portadores de doença de Chagas que recebem benefícios previdenciários e assistenciais no Brasil foram apresentados. Estes achados podem nortear a definição de prioridades de ações de acompanhamento pela atenção primária à saúde, preconizada atualmente para o manejo longitudinal da doença.

## INTRODUCTION

Chagas disease (CD) is a tropical disease with a chronic evolution and high morbidity and mortality^1^. Most infections are asymptomatic, but up to 30% of chronically infected people develop cardiac alterations and up to 10% develop digestive, neurological, or mixed alterations^1,2^.

CD, especially chronic CD with heart involvement, has great disabling potential, removing individuals with productive potential from the labor market^3^. In addition to physical disability, CD patients suffer from psychological impacts, such as fear, stress, anxiety, low self-esteem, depression, and, consequently, they have a low quality of life^1,4,5^.

In recent decades, there has been a decline in mortality due to CD; however, the disease still remains an important cause of death in the country, especially in people over 50 years of age^2,6^. Between 2000 and 2019, 122,291 deaths from CD were recorded, which represented 0.54% of the overall total of deaths recorded in the country in that period^6^. CD was the leading cause of death (76.7%) among neglected tropical diseases in Brazil between 2000 and 2011^7^.

Health care related to CD is related to medium and high complexity services, mainly due to the severity of the cases or due to the lack of adequate targeting/referral of individuals^8,9^. The follow-up of chronic cases by the Primary Health Care (PHC) has recently been recommended as a way to mitigate the social impact and improve the quality of life of patients affected by the disease^8-10^. In social security and social assistance, there has been a reduction in the number of granted benefits in recent years, but the chronicity of the disease and its disabling potential means that many workers and people in situations of social vulnerability are receiving benefits^3^.

In Brazil, there is no precise estimate of the number of people infected/chronically ill by CD. The last national serological survey was conducted from 2001 to 2008 among children up to 5 years of age, from rural areas, and resulted in a prevalence of 0.03%^11^. However, it is estimated that due to the high transmission rates that occurred in the last century, there are currently between 1.9 and 4.6 million people infected by *Trypanosoma cruzi* in the country^1,12^. Regional seroprevalence studies have been carried out recently, such as in highly endemic areas in the Brazilian states of Bahia (prevalence of 4.4%) and Ceará, in urban areas (prevalence of 4.2%), and in rural areas (prevalence of 3.7%)^13-15^.

In 1990, there were an estimated 700 thousand new cases of CD in Latin America. In 2010, this estimate was 29,925 cases^1^. In Brazil, in the period between 2001 and 2018, only 5,184 new cases of CD were registered, with an annual incidence rate of 0.16 per 100 thousand inhabitants/year^16^.

The national mandatory notification of chronic cases was instituted in 2020; nevertheless, due to the incipience of the process, there is still a limitation of the data source of these cases for the construction of indicators^17,18^.

In view of the great epidemiological silence about chronic CD in Brazil, the use of data from Brazilian social security and social assistance becomes very useful as indicators of morbidity and mortality. In this study we analyzed the survival of CD patients who received social security and social assistance benefits in Brazil from 1942 to 2016.

## METHODS

### Study design

This is a retrospective cohort study (open cohort) analyzing the survival of CD patients who are beneficiaries of social security and social assistance in Brazil, in two distinct periods: from the onset of the disease to death and from the onset of work disability to death.

### Context

Sociodemographic data and data on the granting of social security and assistance benefits to individuals with CD were analyzed.

The date of onset of the disease, between January 1942 and October 2016, was estimated and recorded by medical experts from the National Institute of Social Security (*Instituto Nacional do Seguro Social* – INSS). The estimate for this date was based on the reports of the beneficiaries during the expert evaluation on the time of the appearance of the first signs and symptoms related to CD. The estimated date was recorded in the forensic anamnesis. The date of onset of work disability, which corresponded to the date on which the disease incapacitated the beneficiary from performing the work activity, was also recorded by the INSS medical experts.

### Participants

People affected by CD who received social security or assistance benefits during the period from January 1st, 2004 to December 31, 2016.

### Variables

The following independent variables were analyzed: sex (men and women), age group (adults and older adults), period of disease manifestation (1942–1999 and 2000–2016), area of residence (urban or rural), clinical form (cardiac, digestive, and other clinical/unspecified forms), geographic macroregion (South, Southeast, Midwest, North, and Northeast), type of benefit received (assistance and social security), and year of concession (2004 to 2016).

The sex variable was based on biological distinction, as recommended by the *Diretrizes para Equidade de Sexo e Gênero na Pesquisa* (Guidelines for Sex and Gender Equity in Research). In the age group variable, individuals aged between 18 and 59 years were classified as adults and those aged 60 years or older were classified as older adults. All CD classifications according to the Tenth Revision of the International Statistical Classification of Diseases and Related Health Problems (ICD-10) were included in the study, namely: B57 (unspecified CD); B57.0 (Acute Chagas disease with heart involvement); B57.1 (Acute Chagas disease without heart involvement); B57.2 (Chagas disease [chronic] with heart involvement); B57.3 (Chagas disease [chronic] with digestive system involvement); B57.4 (Chagas disease [chronic] with nervous system involvement); B57.5 (Chagas disease [chronic] with other organ involvement); K23.1 (Megaesophagus in Chagas Disease) and K93.1 (Megacolon in Chagas disease). However, when survival was specifically analyzed in relation to the clinical forms of the disease, only the chronic cardiac and digestive forms were considered, as they represent the most prevalent forms and those with the highest morbidity and mortality.

Two dependent variables were used:

1. The time elapsed between the onset of the disease and death (minimum follow-up of 1 month and maximum of 815 months); and
2. Time elapsed between work disability and death (minimum follow-up of 1 month and maximum of 398 months).

### Data source

The data source was the Unified System of Benefits Information of the Brazilian Ministry of Social Security (*Sistema Único de Informações de Benefícios* – SUIBE), with restricted access, developed by the Social Security Technology and Information Company (*Empresa de Tecnologia e Informações da Previdência Social* – DATAPREV)^19^. Data were accessed after a formal request to the central management level of the INSS. The data were made available in an Excel^®^ spreadsheet in January 2017.

### Bias control

One of the premises for the use of Cox regression is that the relationships between the response variable and the independent variables are proportional throughout the analyzed period^20^. To verify this premise, Schoenfeld residual analysis was performed for each of the independent variables.

### Statistical methods

Initially, a descriptive analysis of the data was performed by calculating the absolute and relative frequencies of categorical variables and the medians for numerical variables. The Shapiro-Wilk statistical test was used to verify the normality of the data. For the two dependent variables, failure was deemed as the occurrence of death and censoring, i.e., the individual’s survival at the end of the period considered in this study. The survival curves for the two dependent variables were characterized by the Kaplan-Meier survival curve, while the comparison of these curves according to each independent variable was performed using the Log Rank test^20^. The median duration of each analyzed period was also estimated using the Kaplan-Meier survival curve. All categorical variables were expressed as frequencies and 95% confidence intervals.

To calculate the hazard ratio (HR) in the survival analysis, the Mantel Haenszel method was used^20^. The Cox proportional hazards model was used to establish the relationship between the independent variables and death, by calculating the risks (HR) and their respective 95% confidence intervals and defining the predictor covariates of survival time^20^.

According to Schoenfeld’s, analysis of residuals, for the period “time between the onset of the disease and death,” there was a violation of the proportionality of risks for the variables “clinical form of the disease” and “sex”; and for the period “time between work disability due to the disease and death” there was a violation of the proportionality of risks for the variables “sex,” “period of disease manifestation,” “area of residence,” and “type of benefit.” Thus, the time partition for these variables in the model was carried out, considering that the analysis of residuals showed that the risks were not equal over time^20^.

The chi-square test was used to compare proportions. The Kruskal-Wallis test was used to compare numerical variables.

The level of significance adopted in the study was 5%. All analyses were performed using Stata 15.0 software.

### Ethical considerations

The study was approved by the Human Research Ethics Committee of the Universidade Federal de Uberlândia on May 17, 2016, CAAE: 52527516.4.0000.5152.

## RESULTS

We analyzed data from 32,412 beneficiaries. The sociodemographic and epidemiological characteristics of the beneficiaries are shown in Table 1. For 8,933 (27.6%) beneficiaries, the clinical form of CD was unspecified. Other clinical forms of lower occurrence were observed, namely: 614 (1.9%) benefits were due to acute CD with heart involvement; 136 (0.4%) benefits due to acute CD without heart involvement; 83 (0.3%) benefits due to chronic CD with other organ involvement; and 10 (0.03%) benefits due to chronic CD with nervous system involvement (Table 1).

**Table 1:** Sociodemographic and epidemiological characteristics of patients with Chagas disease who benefit from social security and social assistance in Brazil (n=32,412).

Variables	Frequency % (95%CI)
Sex	
Men	62.7 (62.2–63.2)
Women	37.3 (36.8–37.8)
Age group	
Adults	53.9 (53.3–54.4)
Older adults	46.1 (45.6–46.7)
Clinical form	
Cardiac	57.0 (56.5–57.6)
Digestive	12.8 (12.4–13.2)
Other clinical forms/Unspecified	30.2 (29.7–30.7)
Area of residence	
Urban	67.1 (66.6–67.6)
Rural	32.9 (32.4–33.4)
Geographic macroregion	
North	1.9 (1.7–2.0)
Northeast	30.6 (30.1–31.1)
Midwest	17.8 (17.3–18.2)
Southeast	46.9 (46.4–47.5)
South	2.8 (2.7–3.0)
Period of disease manifestation*	
1942 to 1999	19.4 (19.0–19.9)
2000 to 2016	80.6 (80.1–81.0)
Types of benefits granted	
Assistance	6.7 (6.5–7.0)
Social security	93.3 (93.0–93.5)

95%CI: 95% confidence interval. *n=27,673 beneficiaries.

Of the total number of beneficiaries, 30,234 (93.3%) received social security benefits and only 2,178 (6.7%) received assistance benefits. The most frequent social security benefits were temporary disability allowance (20,553; 63.4%) and permanent disability retirement (9,640; 29.7%). Among the social security beneficiaries, the majority were men (19,294; 63.8%), living in urban areas (19,574; 64.8%), and adults (16,362; 54.1%). As for assistance benefits, assistance support was only granted to people with disabilities (2,178; 6.7%) (Table 1). Among the beneficiaries of social assistance, the majority were women (1,145; 52.6%), living in urban areas (2,167; 99.5%), and adults (1,161; 50.6%).

Most of the beneficiaries in this study were adults (17,470; 53.9%), and 92.3% of the beneficiaries in this age group were 40 years old or older. The median age, considering all beneficiaries included in this study, was 59 years. Benefit concessions were mainly concentrated in the period between 2004 and 2009 (20,435; 63.1%) (Table 1).

**Figure 1:**
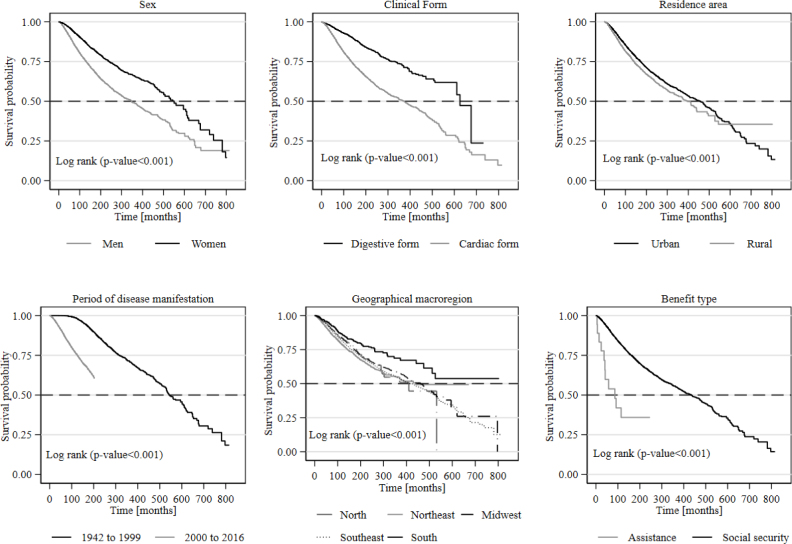
Kaplan-Meier survival curves of the time (in months) between the onset of Chagas disease and death, according to sociodemographic and epidemiological variables. Brazil, 1942–2016.

**Figure 2:**
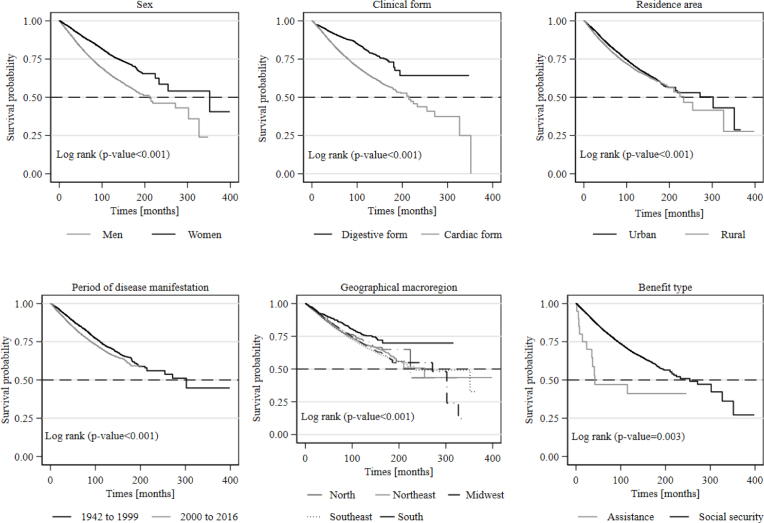
Kaplan-Meier survival curves of the time (in months) between the onset of work disability due to Chagas disease and death, according to sociodemographic and epidemiological variables. Brazil, 1942–2016.

In the Kaplan-Meier analyses related to the periods between the onset of the disease and death and between the onset of work disability and death, we verified higher survival among women beneficiaries who had the digestive form of the disease, residents of urban areas, who manifested the disease in the period from 1942 to 1999, and among those who received social security benefits. In both analyzed periods, the South, Northeast, and Midwest macroregions had the longest survival times (Figures 1 and 2).

Cox regression was performed separately for each analyzed period. In both periods, the variables associated with higher survival were being a woman and receiving social security benefits. The heart form of the disease, manifestation of the disease between 2000 and 2016, and living in a rural area were associated with lower survival (Table 2).

**Table 2:** Hazard ratio and 95% confidence interval of the variables associated with the survival of beneficiaries of Brazilian social security and social assistance affected by Chagas disease, in crude and adjusted analysis by Cox regression (n=32,412).

Variable	Time between disease onset and death		Time between work disability and death	
	Crude hazard ratio (95%CI)	Adjusted hazard ratio (95%CI)	Crude hazard ratio (95%CI)	Adjusted hazard ratio (95%CI)
Sex				
Men	Ref	Ref	Ref	Ref
Women	0.41 (0.40;0.50)	0.54 (0.43;0.53)	0.50 (0.42;0.53)	0.51 (0.41;0.52)
Clinical form of the disease*				
Digestive	Ref	Ref	Ref	Ref
Cardiac	2.93 (2.51;3.31)	2.64 (2.23;3.12)	2.02 (1.83;2.23)	1.95 (1.83;2.13)
Area				
Urban	Ref	Ref	Ref	Ref
Rural	1.42 (1.32;1.52)	1.23 (1.14;1.21)	1.30 (1.24;1.43)	1.31 (1.21;1.54)
Geographic microregion				
North	Ref	Ref	Ref	Ref
Northeast	1.10 (0.92;1.43)	1.12 (0.81;1.41)	1.12 (0.92;1.33)	1.01 (0.84;1.42)
Midwest	0.91 (0.72;1.11)	0.93 (0.73;1.21)	1.14 (0.91;1.31)	1.03 (0.84;1.44)
Southeast	0.91 (0.71;1.21)	1.11 (0.74;1.33)	1.23 (1.00;1.4)	1.24 (0.91;1.52)
South	0.63 (0.44;0.83)	0.62 (0.43;1.02)	0.81 (0.63;1.01)	0.81 (0.63;1.72)
Period of disease manifestation*				
1942 to 1999	Ref	Ref	Ref	Ref
2000 to 2016	5.01 (4.62;5.54)	5.32 (4.74;5.93)	1.62 (1.43;1.72)	1.53 (1.33;1.71)
Benefit type				
Assistance	Ref	Ref	Ref	Ref
Social security	0.22 (0.12;0.42)	0.13 (0.11;0.23)	0.12 (0.13;0.34)	0.24 (0.14;0.45)

95%CI: 95% confidence interval; Ref: denominator reference. *n=22,634; ^†^n=27,673.

The percentage of deaths in the groups that manifested the disease between the years 1942–1999 and between the years 2000–2016 were quite similar: 25.3 and 25.7%, respectively (p=0.563). However, the median age of older cases (1942–1999) was higher (61 years) when compared to more recent cases (2000–2016) (57 years) (p<0.001).

## DISCUSSION

The sample of Brazilian social welfare beneficiaries affected by CD was mainly composed of men, over 40 years of age, living in urban areas in the Southeast region, with chronic CD with heart involvement, and receiving social security benefits. The highest percentage of benefit concessions was in the 2004–2009 period. The highest survival in both analyzed periods was associated with women, manifestation of the disease between 1942–1999, digestive clinical form of the disease, living in urban areas, and receiving social security benefits.

Social security coverage for men in the General Social Security System (*Regime Geral da Previdência Social* – RGPS), the system analyzed in this study, has always been higher when compared to the coverage for women, even after the increase in women’s participation in the labor market as of the 1990s^21^. Women still represent the majority among workers without a formal employment relationship, unpaid workers, and workers in production for their own consumption^21^. This partly explains the fact that most of the individuals in the study were men. In addition, overall, CD has a more severe and disabling evolution in men, who traditionally seek healthcare services less for diagnosis and appropriate treatment^3^.

Currently, most new cases of CD are related to oral transmission through the ingestion of contaminated food or vector transmission outside the household, but in a much smaller number compared to the incidences of the past^2^. This fact was reflected in the age profile of the beneficiaries, individuals over 40 years of age, infected in previous decades in rural areas, and who are currently experiencing the clinical manifestations of the disease. The lower percentage of concessions in the period between 2010 and 2016 is also related to the lower incidence of CD, associated with high mortality among older adults, factors that influenced the decrease in the prevalence of the disease^6^.

Most CD patients migrated to cities, establishing a new epidemiological context, chronic CD with a higher prevalence in urban areas, as demonstrated in this study^1^.

In absolute numbers, the Southeast macroregion was the one with the highest frequency of beneficiaries. In addition to being the most populous and urbanized macroregion in the country, it has higher human development indexes, making its resident population more apt to contribute to social security and more informed about existing and pertinent welfare rights^3^. The macroregion also encompasses two historically very important states for CD transmission: Minas Gerais and São Paulo^1,3^.

The largest number of benefits were granted to individuals affected by the cardiac form of CD. Chronic CD with heart involvement is the most frequent clinical form with great disabling potential, especially for workers who exert great physical efforts in their work activities^3,22^.

The individuals analyzed in this study received mainly social security benefits, i.e., they participated in the country’s labor force before the occurrence of temporary or permanent work disability. Conversely, in studies carried out in Brazil on social welfare and AIDS, researchers shown that among 99,369 beneficiaries, 26.5% received assistance and 51% were unemployed^23,24^.

Women were associated with longer survival both from the onset of the disease and in relation to the onset of work disability. In fact, it has been documented in other studies that CD mortality among women is lower^6,7,25^. Environmental, behavioral, and sociocultural factors influence this difference. Work activities that require much physical effort, for example, are usually performed by men and are related to a poor prognosis of cardiac CD^26^. The greater demand for healthcare services and the concern with self-care, which are closely related to women, also contribute to avoiding more severe cases of the disease and, consequently, death in this group^27^.

Regarding the period of manifestation, CD patients who manifested the disease between 1942 and 1999, despite being a minority in the study, had higher survival. This finding, at first counterintuitive, can be explained by the formula used to calculate the hazard ratio, a formula that considers the number of outcomes (deaths) in the numerator and the follow-up time of individuals in the denominator of each group. As observed in the study, the percentage of deaths in both groups was very similar, even though the group of older cases was composed of older individuals who manifested the disease in periods when technology and access to health services were more precarious. Conversely, obviously, the follow-up time of these cases was considerably longer when compared to the group that manifested the disease recently (2000–2016). Thus, in the calculation of HR, a higher risk was observed in this group. This finding may indicate a worsening in the management of CD patients in healthcare services, influenced by the decrease in the incidence of the disease in recent decades^1^. Indeed, there is a great epidemiological silence in Brazil in relation to chronic cases of CD, and only recently has this been changing with the mandatory notification of chronic cases and the recommendation of follow-up of patients by PHC^8-10,17,28^.

In addition to the loss of physical health, the diagnosis of CD and the finding of work disability lead to cognitive and psychosocial impairments that significantly alter the quality of life of patients and influence the evolution of the disease^4,29^. In this sense, it has been recommended that PHC be a protagonist in the screening and longitudinal follow-up of CD patients. With the protagonism of this level of health care, groups that are more vulnerable to the disease, such as men, can be reached, greatly contributing to the quality of life of individuals, including increasing survival^8-10^.

Survival among patients with CD with the digestive clinical form was considerably higher when compared with those affected by the cardiac form. Indeed, chronic CD with heart involvement is associated with higher mortality^3,6,8,9,25^. However, in a study on CD mortality between 2000 and 2010, an increase in mortality related to the digestive system was observed^25^. One of the explanations for this finding was the adoption of specific codes for CD with digestive involvement in the 10th version of the ICD. Despite the longer survival, more advanced cases of the “mega” forms present a high degree of morbidity and considerable loss of quality of life, with disorders, such as dysphagia, regurgitation, fear of eating and malnutrition, being frequent among individuals with megaesophagus and severe constipation, presence of fecaloma and volvulus in cases of megacolon^30^.

The migration of individuals infected with *T. cruzi* to urban areas has been intense in recent decades^31,32^. In Brazil, about 70 to 75% of individuals with CD live in urban areas^31^. During the migration process, the peripheral areas of the municipalities were the main destination of these individuals, often in precarious living conditions^31,32^. Nevertheless, access to healthcare services, which is mainly available in the public sector, has certainly been improved in the urban context^32^. This improvement may have influenced the longer survival of individuals living in urban areas. Furthermore, rural work, typically associated with manual labor, contributed to more frequent death outcomes in these areas^22,26^.

Higher survival was observed among individuals who received social security benefits. To receive these benefits, the contribution is mandatory^3^. This shows that, in most cases, these individuals were in the labor market, in better and more stable socioeconomic conditions, when compared to those of individuals who received assistance benefits. They present a situation of significant vulnerability, characterized by the inability to have the means to provide for their own maintenance, incapacity to work caused by the disease, and precarious family conditions (income per person in the family group less than 1/4 of the minimum wage). The social vulnerability to which these individuals are subjected, mainly due to income issues and inequality of access to goods and services, is reflected in the lower survival presented by them in this study.

The use of the RGPS database imposed an important limitation on the study. The analyzed sample included only individuals with CD enrolled in the social security system of this regime or those who met the basic requirements to be entitled to assistance benefits. This condition led to an underestimation of the number of workers affected by the disease, as they did not include those individuals in the informal sector or those who were subject to their own social security regimes. Another important limitation refers to the timeliness of data, as this study has information on CD in social welfare only up to the year 2016.

Inequalities in the survival of CD patients were observed in a large Brazilian cohort, both from the onset of the disease and from work disability. In both outcomes, the following categories of variables were predictors of longer survival: being a woman and receiving social security benefits. Lower survival was associated with the cardiac form of the disease, living in a rural area, and manifestation of signs and symptoms of the disease between 2000 and 2016. These findings can guide the definition of priorities for follow-up actions by the PHC, currently recommended for the longitudinal management of the disease.
